# Human Herpesvirus 6 Encephalitis in an Immunocompetent Elderly Adult: A Case Report

**DOI:** 10.7759/cureus.100066

**Published:** 2025-12-25

**Authors:** Bruna Rodrigues Barbosa, Joana Ferreira, Inês Bonito, Inês P Carvalho, Ana Paula Pona Pisco

**Affiliations:** 1 Internal Medicine, Unidade Local de Saúde Arco Ribeirinho - Centro Hospitalar Barreiro Montijo, Barreiro, PRT

**Keywords:** diplopia, gait ataxia, geriatric patient, hhv-6 encephalitis, immunocompetent adult

## Abstract

Encephalitis is an inflammatory disorder of the brain parenchyma associated with significant neurological morbidity. Viral infections represent the most frequent etiologies, and although human herpesvirus 6 (HHV-6) is a ubiquitous childhood pathogen, HHV-6 encephalitis in immunocompetent adults is rare. Diagnostic interpretation is challenging because HHV-6 DNA may also be detected in individuals with chromosomally integrated HHV-6. We present the case of an 84-year-old man with well-controlled type 2 diabetes and a history of treated solid-organ malignancies, but no evidence of immune dysfunction or immunosuppressive therapy, who developed progressive gait ataxia, intermittent diplopia, and systemic inflammation. Cerebrospinal fluid (CSF) polymerase chain reaction (PCR) tested positive for HHV-6. The patient achieved complete neurological recovery following antiviral therapy. This case emphasizes the need to think about HHV-6 infection in older adults with unexplained neurological decline and underscores diagnostic and therapeutic complexities, particularly regarding the differentiation between viral reactivation and chromosomal integration.

## Introduction

Encephalitis is defined as inflammation of the brain parenchyma accompanied by neurological dysfunction [1]. Viral infections account for most cases, including herpes simplex virus, enteroviruses, and arboviruses [2]. Human herpesvirus 6 (HHV-6), first identified in 1986 in patients with lymphoproliferative disorders, consists of two distinct species: HHV-6A and HHV-6B [3,4]. HHV-6B causes exanthema subitum during early childhood, after which the virus remains latent with the potential for reactivation, particularly in immunocompromised hosts such as hematopoietic stem cell transplant recipients [5].

Although HHV-6 encephalitis is well recognized in immunocompromised individuals, several reports document clinically significant CNS involvement in immunocompetent adults, with presentations ranging from encephalopathy to cerebellar ataxia and focal neurological deficits [6-12]. A positive polymerase chain reaction (PCR) in the cerebrospinal fluid (CSF) is frequently the first step in the diagnosis of HHV-6 encephalitis, although interpreting that result might be challenging. About 1% of people naturally carry HHV-6 integrated into their DNA, which means they can show high viral levels even when the virus isn’t actually active [13]. In the absence of strong clinical trial data, treatment decisions for immunocompetent adults still rely largely on observational studies and expert opinion. Antiviral therapy with ganciclovir, valganciclovir, or foscarnet is frequently considered in progressive or severe presentations [14-16].

We describe a case of HHV-6 encephalitis in an elderly immunocompetent patient presenting predominantly with gait ataxia and ocular symptoms, who improved significantly with antiviral therapy. Accordingly, this report aims to delineate the diagnostic reasoning required to distinguish active HHV-6 infection from chromosomally integrated HHV-6 in an elderly patient with subtle, subacute manifestations and to discuss the therapeutic decision-making process.

## Case presentation

An 84-year-old man with a known history of hypertension and type 2 diabetes (HbA1c 6.1%) presented to the emergency department (ED) after the sudden onset of dizziness and malaise, nearly resulting in syncope. His vision had become blurred as well. He had previously undergone a colectomy for colorectal cancer 10 years prior to the presentation and a transurethral bladder resection one year earlier. Despite these comorbidities, he had no evidence of active malignancy or immune dysfunction and was not receiving immunosuppressive therapy at the time of presentation. He lived with his wife and remained active and independent. According to both of them, he had been slowing down over the past few months, with several falls that they initially attributed to age and fatigue, although he had never fully recovered his usual balance. Early monitoring suggested bradycardia, but the ECG was normal. He improved slightly with symptomatic medication and returned home, though not entirely well.

Over the coming days, however, the symptoms returned and gradually worsened. Five days following initial presentation, he presented to the ED again because he felt weaker in his legs and increasingly unsteady when trying to stand or walk. The blurred vision persisted but improved when he covered one eye, something he had noticed at home. During the clinical interview, he and his wife explained that he had been losing stability for months, which made the current deterioration more concerning.

On admission, he was fully oriented, calm, and cooperative. His vital signs were stable; however, he presented a tympanic temperature of 38°C. Neurologically, he had a mild ptosis of the left eyelid, with no associated diplopia, ophthalmoparesis, or nystagmus, and no other cranial nerve abnormalities. Muscle strength was preserved; however, the patient could barely stand without support. Gait examination revealed an ataxic gait that was slightly wide-based, with reduced step stability, external rotation of the feet, and a tendency to drag the right leg, which the patient attributed to pelvic-level pain. There was no truncal ataxia. Finger-to-nose testing demonstrated mild dysmetria, while heel-to-shin testing showed no dysmetria. Deep tendon reflexes and sensation were normal.

The laboratory test results over a period of time are summarized in Table 1, where the patient’s progression can be observed.

**Table 1:** The evolution of the patient's laboratory results. * Herpes simplex virus type 1, herpes simplex virus type 2, cytomegalovirus, varicella-zoster virus, enterovirus, human parechovirus, *Neisseria meningitidis*, *Streptococcus pneumoniae*, *Streptococcus agalactiae*, *Listeria monocytogenes*, *Escherichia coli *K1, *Haemophilus influenzae*, and *Cryptococcus neoformans/gattii* not detected. eGFR: estimated glomerular filtration rate; TSH: thyroid-stimulating hormone; Ag HBS: hepatitis B surface antigen; Ac HBs: antibody to hepatitis B surface antigen; Ac. anti-Trep.: antibodies against *Treponema pallidum*; PCR: polymerase chain reaction; VDRL: Venereal Disease Research Laboratory

Laboratory tests	At Admission (second presentation)	Hospital Day 2	Hospital Day 3 (lumbar puncture)	Hospital Day 5	Hospital Day 6	Hospital Day 12	Reference range
Hemoglobin (g/dL)	13.6	-	13.3	13.2	13	12	13-17
White blood cell count (×10⁹/L)	8.9	-	8.2	8.3	8.9	5.5	3.9-10.2
Neutrophils (%)	68.2	-	73.4	68.8	76.8	69.3	40-75
Platelets (×10⁹/L)	176	-	179	181	188	264	150-400
International normalized ratio	-	-	1.32	-	-	-	0.8 - 1.2
Glucose (mg/dL)	102	-	-	80	90	105	70-105
Urea (mg/dL)	67	-	40	41	37	35	10 - 50
Creatinine (mg/dL)	1.13	-	0.91	0.79	0.78	0.82	0.73 - 1.18
eGFR (mL/min/1.73 m²)	59	-	77	82	83	81	>90
Sodium (mmol/L)	138	-	139	142	142	141	136-146
Potassium (mmol/L)	3.3	-	3.5	3.5	3.5	5	3.5-5.1
Chloride (mmol/L)	102	-	103	105	103	106	101-109
Magnesium (mg/dL)	2.2	-	2.1	-	-	-	1.59 - 2.56
Total bilirubin (mg/dL)	-	-	1.2	-	-	0.4	< 1.2
Direct bilirubin (mg/dL)	-	-	0.4	-	-	-	0.1 - 0.5
Aspartate aminotransferase (U/L)	18	-	14	14	24	61	<34
Alanine aminotransferase (U/L)	17	-	12	10	20	85	<45
Gamma-glutamyl transferase (GGT) (U/L)	-	-	37	-	-	-	< 55
Alkaline phosphatase (U/L)	-	-	74	-	-	101	50 - 116
C-reactive protein (mg/L)	180.3	-	244.5	266.2	293	34.9	<5
TSH (uUI/mL)	1.57	-	-	-	-	-	0.36 - 4.94
Hepatite C IgG; HIV I and II; Ag HBS; Ac HBs; Ac. anti-Trep. pallidum (IgG+IgM)	-	-	-	Negative	-	-	-
Respiratory viral panel	-	Negative	-	-	-	-	-
Urinalysis	-	Isolated glycosuria	-	-	Glycosuria and hematuria	-	-
Blood cultures	-	Negative	-	-	Negative	-	-
Urine culture					Negative	-	-
PCR for neurotropic pathogens in CSF	-	-	Human herpesvirus type 6 detected *	-	-	-	-
CSF microbiology	-	-	Negative	-	-	-	-
VDRL in CSF	-	-	Non-reactive	-	-	-	-
Adenosine deaminase in CSF (U/L)	-	-	0.3	-	-	-	0 - 9

**Figure 1:**
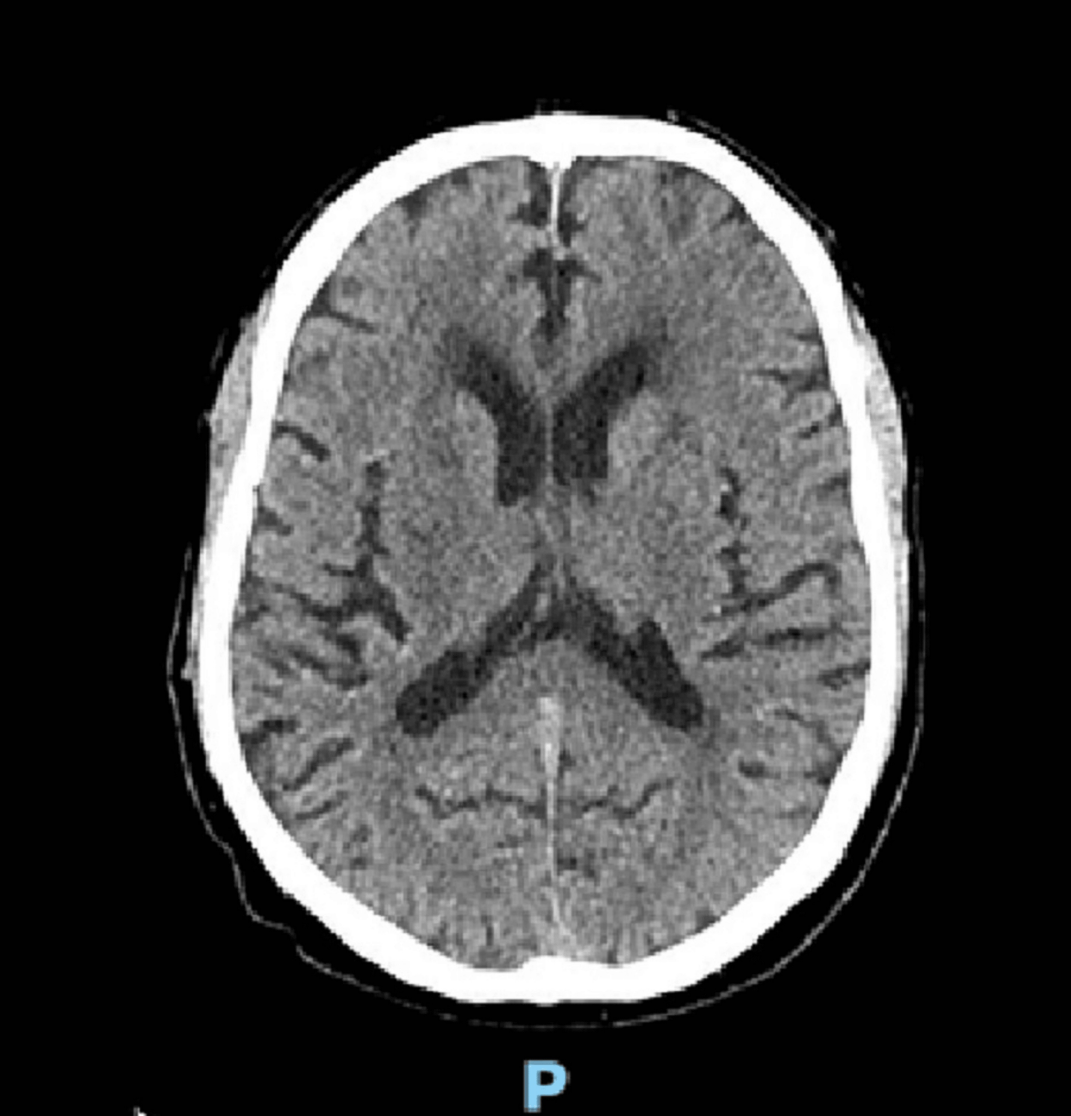
CT of the brain: no acute lesions.

A computed tomography (CT) scan of the brain showed no acute lesions, only age-related changes such as mild cortical atrophy and leukoaraiosis (Figure 1).

**Figure 2:**
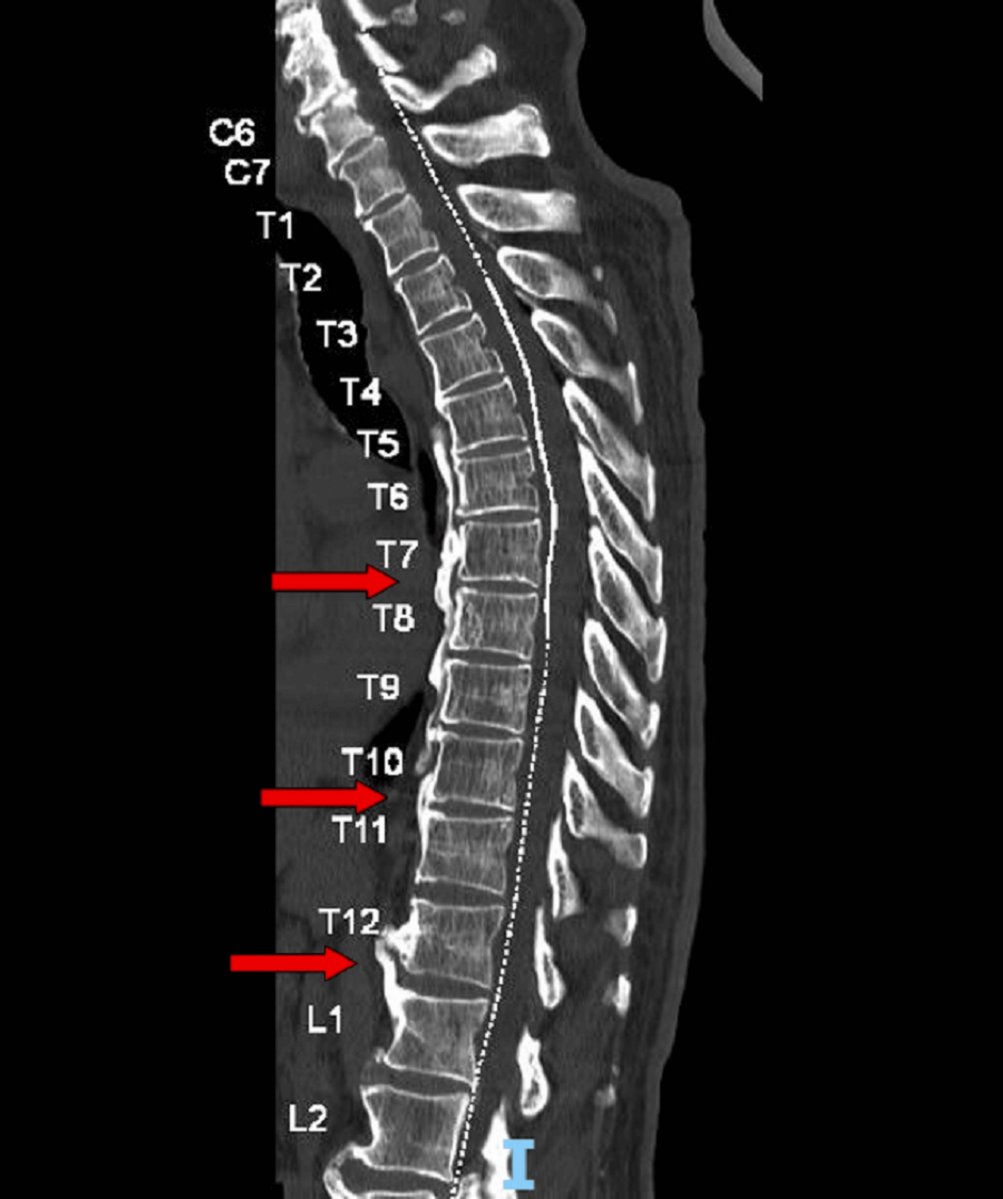
Spinal CT showing findings compatible with DISH. A mild dextroconvex scoliotic alignment is present, without fractures, lytic lesions, or significant canal or foraminal stenosis. Red arrows:  DISH more pronounced in the mid- and lower-thoracic segments, with anterior marginal osteophytes and straightening of the normal curvature. DISH: diffuse idiopathic skeletal hyperostosis

In the context of imbalance associated with gait ataxia and low back pain, a spinal CT scan was obtained, which revealed degenerative disease, including a C7 listhesis and some canal narrowing, at this level, which did not convincingly explain the acute picture. Thoracic spine CT suggested diffuse idiopathic skeletal hyperostosis (DISH) but no fractures or nerve compression (Figure 2).

Given the persistent unsteadiness and rising inflammatory markers, a lumbar puncture was performed two days after admission. The CSF was clear, with only two white blood cells per mm³, normal glucose, and slightly elevated protein. The surprising finding came from PCR testing, which detected HHV-6 DNA in the CSF.

The patient began treatment with valganciclovir (10 days, 900 mg/day). Over the following days, the fever resolved, and his neurological symptoms began to improve. He regained confidence in his ability to walk as his balance gradually improved and the visual disturbance vanished. By the time he was discharged, he was already able to walk on his own again, without the instability that had led him to the hospital, although with a slight imbalance in his gait.

**Figure 3:**
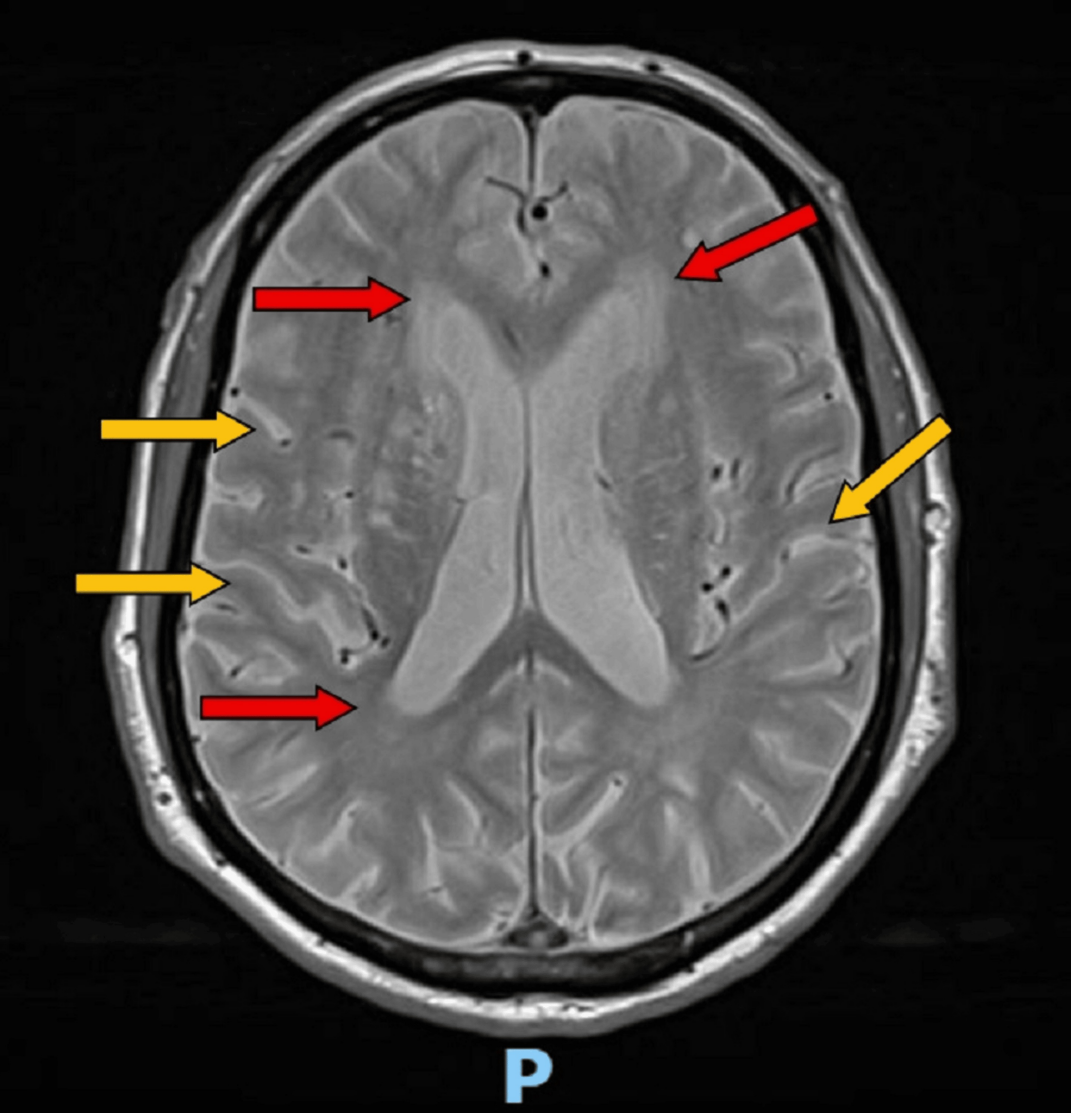
Brain MRI T2 shows no acute or space-occupying lesions. Red arrows: chronic microangiopathic white-matter disease with multiple small hemosiderin foci (likely hypertensive in origin); Yellow arrows : mild cortico-subcortical atrophy, more pronounced in the temporal and parietal regions.

During the admission, he developed arthritis of the left knee and conjunctivitis, both managed without complications. At discharge, he resumed his regular medications and was referred for follow-up in internal medicine. A brain magnetic resonance imaging (MRI) was requested, and he was advised to return to the emergency department if any of his previous symptoms resurfaced. The MRI (Figure 3) was later reviewed at the post-discharge follow-up visit in the Internal Medicine clinic. At the time of discharge, the patient still had a slight gait imbalance, but by the follow-up appointment, he was fully recovered and no longer had any neurological symptoms.

## Discussion

HHV-6 encephalitis in immunocompetent adults is an uncommon but increasingly recognized condition. Recent reviews indicate that although the majority of HHV-6 CNS disease occurs in immunocompromised patients, true encephalitis in healthy adults is likely underdiagnosed due to variability in clinical presentation and the complexity of interpreting PCR results [3,17,18]. Most adults acquire HHV-6 during childhood, and reactivation rather than primary infection is believed to account for most late presentations [13,17].

One of the most significant challenges in diagnosing HHV-6 is telling the difference between an active infection and chromosomally integrated HHV-6 (ciHHV-6) [13,17], where the entire viral genome is inherited and present in all nucleated cells. Because of this, people with ciHHV-6 can show high levels of HHV-6 DNA in both blood and CSF even when the virus isn’t actually active [17,19]. Current diagnostic recommendations therefore stress the need to look at the whole clinical picture, how the patient is presenting, how viral loads evolve, and, whenever possible, to use confirmatory tests like hair-follicle PCR to help identify chromosomal integration [17,19].

In this case, we considered the possibility of ciHHV-6 from the start. Confirmatory testing, like hair follicle PCR, wasn’t available in our setting. The positive CSF PCR for HHV-6, together with herpesvirus detected in the CSF, was therefore interpreted as evidence of active infection based on several factors: the patient’s subacute but steadily worsening neurological condition, the presence of systemic inflammatory signs, the lack of alternative infectious, autoimmune, or structural causes after a thorough evaluation, and the overall severity of the presentation. The sudden neurological decline, fever, and inflammatory response, along with the clear improvement after starting antiviral therapy, strongly supported a diagnosis of true active HHV-6 encephalitis.

CSF results can be subtle, and many published cases, especially in older adults, show little or no pleocytosis, which is precisely what we observed here [6,7]. Neuroimaging findings vary considerably, ranging from normal studies to mesial temporal involvement, and absence of abnormalities does not exclude the diagnosis [8].

Choosing the right treatment can be difficult because there are no strong data from randomized trials to guide decisions. In practice, ganciclovir and foscarnet are the drugs used most often, while valganciclovir is a practical oral option for patients who are stable enough to take it [14-16]. Although resistance mutations, particularly in the *U69* gene, have been documented [16], most immunocompetent patients treated promptly demonstrate substantial improvement. Observational data suggest that early antiviral therapy may improve outcomes, although high-quality evidence remains limited [18]. Adults who are immunocompetent typically recover more quickly than transplant recipients, yet some may still experience persistent neurological symptoms [3].

This case brings several key points into focus. HHV-6 should be considered in the differential diagnosis of older patients who present with unexplained cerebellar signs or cranial nerve abnormalities. A positive CSF PCR result also needs to be interpreted with care, particularly when chromosomally integrated HHV-6 is a possibility. Finally, early antiviral therapy can support meaningful recovery, even in individuals without any underlying immunosuppression. Larger multicenter studies are still needed to better define the most effective diagnostic approaches, determine when antiviral treatment is truly warranted, and clarify long-term patient outcomes.

## Conclusions

HHV-6 encephalitis should be considered in elderly immunocompetent adults who develop unexplained neurological symptoms. A positive CSF PCR needs to be interpreted with caution, as it may reflect either active infection or chromosomal integration. In our patient, starting antiviral treatment early was followed by a full recovery. However, this is just one case, so we cannot draw firm conclusions about the effectiveness of treatment, and more studies are needed to understand when and in whom therapy is truly beneficial. Recognizing the less typical presentations of HHV-6 and improving how we diagnose it are important steps toward better outcomes.
